# Comparison of percutaneous and transarterial fiducial marker placement before proton therapy for pancreatic cancer

**DOI:** 10.1093/jrr/rrag032

**Published:** 2026-05-14

**Authors:** Jumpei Shoji, Kengo Ohta, Kazushi Suzuki, Akihiro Horibe, Motoki Hatano, Kazuma Murai, Koichiro Nakajima, Hiromitsu Iwata, Hiroyuki Ogino, Akio Hiwatashi

**Affiliations:** Nagoya City University West Medical Center, Diagnostic Radiology Department, 462-8508, 1-1-1, Hirate-cho, kita-ku, Nagoya, Japan; Radiology Department, Nagoya City University Graduate School of Medical Sciences, 467-8601, 1, Kawasumi, Mizuho-cho, Mizuho-ku, Nagoya, Japan; Radiology Department, Nagoya City University Graduate School of Medical Sciences, 467-8601, 1, Kawasumi, Mizuho-cho, Mizuho-ku, Nagoya, Japan; Radiology Department, Nagoya City University Graduate School of Medical Sciences, 467-8601, 1, Kawasumi, Mizuho-cho, Mizuho-ku, Nagoya, Japan; Nagoya City University West Medical Center, Diagnostic Radiology Department, 462-8508, 1-1-1, Hirate-cho, kita-ku, Nagoya, Japan; Diagnostic Radiology Department, Midori Municipal Hospital, 458-0037, 1-77, Siomigaoka, Midori-ku, Nagoya, Japan; Nagoya City University West Medical Center, Diagnostic Radiology Department, 462-8508, 1-1-1, Hirate-cho, kita-ku, Nagoya, Japan; Radiology Department, Nagoya City University Graduate School of Medical Sciences, 467-8601, 1, Kawasumi, Mizuho-cho, Mizuho-ku, Nagoya, Japan; Nagoya Proton Therapy Center, Radiation Oncology Department, Nagoya City University West Medical Center, 462-8508, 1-1-1, Hirate-cho, kita-ku, Nagoya, Japan; Nagoya Proton Therapy Center, Radiation Oncology Department, Nagoya City University West Medical Center, 462-8508, 1-1-1, Hirate-cho, kita-ku, Nagoya, Japan; Nagoya Proton Therapy Center, Radiation Oncology Department, Nagoya City University West Medical Center, 462-8508, 1-1-1, Hirate-cho, kita-ku, Nagoya, Japan; Radiology Department, Nagoya City University Graduate School of Medical Sciences, 467-8601, 1, Kawasumi, Mizuho-cho, Mizuho-ku, Nagoya, Japan

**Keywords:** fiducial marker, percutaneous placement, pancreatic cancer, proton therapy

## Abstract

**Purpose:**

This study aimed to evaluate the feasibility and safety of percutaneous fiducial marker placement using a 25G needle compared with transarterial placement before proton therapy for pancreatic cancer.

**Materials and methods:**

Patients who underwent fiducial marker placement from 2013 to 2023 were retrospectively reviewed. For percutaneous placement, a 25G needle was used to place markers in or around the tumor under computed tomography (CT) guidance. For transarterial placement, a metal coil was placed as a fiducial marker in a blood vessel near the tumor using a microcatheter. Technical success was defined as the reliable use of the placed fiducial marker throughout the entire proton therapy course. Major complications were defined as grade 3 or higher. Each outcome was compared using Fisher’s exact test or the Mann–Whitney U test.

**Results:**

Forty-nine and 60 patients underwent percutaneous and transarterial fiducial marker placement, respectively. The technical success rates were 89.8 and 90.0% for percutaneous and transarterial placement, respectively, with no statistically significant difference between them (*P* > 0.99). There were no major complications in either case. The median procedure time for percutaneous placement was significantly shorter than that for transarterial placement (3.3 vs 40.0 min; *P* < 0.001). The median distance between the marker and the tumor was shorter with percutaneous placement than with transarterial placement (0.0 vs 10.0 mm; *P* = 0.005).

**Conclusion:**

Percutaneous placement of fiducial markers for pancreatic cancer proton therapy using a 25G needle could be performed faster than transarterial placement with a high success rate.

## INTRODUCTION

Pancreatic cancer, whose global incidence has increased significantly in recent decades, is expected to remain the main cause of cancer-related deaths in the future [1]. Risk factors for pancreatic cancer include smoking, excessive alcohol consumption, obesity, diabetes, chronic pancreatitis, genetics and human microorganisms [2]. The number of people with risk factors, particularly obesity and diabetes, is on the rise due to recent lifestyle changes. In addition, advances in medical science have improved diagnostic capabilities, leading to increased pancreatic cancer detection rates and a consequent increase in the age-standardized incidence rate worldwide [1].

The mainstay of pancreatic cancer treatment is surgical resection and adjuvant chemotherapy [3]; however, some patients cannot undergo surgery due to the tumor being too advanced, low surgical tolerance, or social issues. In recent years, radiation therapy for pancreatic cancer, including proton therapy, has attracted attention. This topic has been the subject of much research, including neoadjuvant chemoradiation and even definitive treatment of unresectable cancer [4].

In radiation therapy for locally advanced pancreatic cancer, it has been reported that placing and tracking fiducial markers can refine the irradiation range, reduce the incidence of complications and shorten the treatment period [5, 6]. Because the pancreas and its surrounding organs such as the duodenum move due to peristaltic motion, adverse events such as late gastrointestinal toxicity may occur even if the initial dose criteria are met. By placing fiducial markers, the locational relationship between the tumor and surrounding organs can be corrected for each treatment [7].

Reported methods for placing fiducial markers include transarterial placement using a catheter, ultrasound endoscopy and placement via laparotomy; however, all these methods have problems such as being complicated, time-consuming, expensive and highly invasive [8–11]. Since 2016, we have performed percutaneous fiducial marker placement. The size of the needle used for placement was 25G, which is thin and expected to reduce the incidence of complications. Therefore, this study aimed to evaluate the feasibility and safety of percutaneous fiducial marker placement using a 25G needle compared with transarterial placement before proton therapy for pancreatic cancer.

## MATERIALS AND METHODS

This retrospective study was approved by the institutional review board of our institution. Because this was a retrospective study, we posted the study protocol on our website and ensured that study subjects could opt out if they wished.

Treatment for pancreatic cancer was based on the most standard guidelines in our country [12]. For locally advanced unresectable pancreatic cancer, chemotherapy alone or chemoradiotherapy is recommended. However, for patients who are resistant to these treatments or for whom conventional photon beam therapy is expected to cause severe side effects in surrounding organs, proton therapy, which has shown improved therapeutic outcomes in recent years, may also be considered [4]. Based on these findings, the decision to use proton therapy was made at a conference involving interventional radiologists, radiation oncologists, gastroenterologists and gastrointestinal surgeons.

In this study, we included patients who had fiducial marker placement before proton therapy for the primary pancreatic cancer lesion between October 2013 and December 2023. Patients who had their positions corrected by bile duct stent matching, those who were irradiated without marker placement because the amount of tumor movement was judged to be small, those whose lymph node metastases or distant metastases were irradiated, and those who could not place a marker due to bleeding tendencies or signs of infection were excluded. We also excluded patients who had a marker placed but did not undergo proton therapy for some reason. The method of placement (percutaneous or transarterial) was determined based on the opinion of the interventional radiologists and the patient’s wishes. Initially, percutaneous placement was not covered by insurance, so transarterial placement was the main method used. The number of cases using percutaneous placement was gradually increased while confirming its safety. After percutaneous placement became covered by insurance, percutaneous placement was eventually used in most cases, except for cases where it was difficult to secure a puncture route.

### Fiducial marker placement

Marker placement was performed by five interventional radiology specialists and one diagnostic radiologist with 7–28 years of relevant work experience. For percutaneous fiducial marker placement, the puncture point was determined using angio-CT unit (SOMATOM Emotion 16 and Artis zee TA, Siemens Healthineers AG, Erlangen, Germany). A 25G needle (Gold Anchor GA150-10, Naslund Medical AB, Huddinge, Sweden. Needle: 25G × 152 mm, Marker: *φ*0.28 × 10 mm) was used. This fine needle can place the fiducial marker in a folded, straight, or tadpole-shaped form [13]. In this study, we intended to place the marker in a folded form. Before the procedure, we referred to CT and Magnetic Resonance Imaging (MRI) and decided on the location to place the fiducial marker alongside the radiation oncologists. Our general plan was to place at least one marker inside or near the tumor from the dorsal side. If puncturing from the dorsal side was difficult, puncturing from the ventral side was considered via the abdominal fat and/or liver or stomach. The puncture was performed without anesthesia. While the patients held their breath, the needle was advanced 1–3 cm at a time under intermittent CT fluoroscopic guidance. The fiducial marker was placed in the tumor or pancreatic parenchyma or surrounding tissue. For percutaneous placement, the marker was intended to be foldable for ease of placement and visibility during positioning. Thereafter, CT was performed to confirm the fiducial marker’s location and complications such as bleeding. If the marker was not placed at the planned location, we placed an additional one.

For transarterial fiducial marker placement, all approaches were made from the right femoral artery. A 4Fr 25 cm sheath (Super Sheath, Medikit, Tokyo, Japan) was inserted into the right femoral artery, and a 4Fr Shepard Hook type catheter (SHK-N18, Medikit, Tokyo, Japan) was advanced to the celiac artery or the superior mesenteric artery. The artery near the pancreatic tumor was selected with a 1.8/2.7Fr microcatheter (Carry Gaia, UTM, Aichi, Japan). A pushable embolization platinum microcoil (MWCE-18S-0.5-0-HILAL, Cook Medical, Indiana, USA. 0.018-inch diameter, 5 mm long, straight form) was placed as a fiducial marker. The location of the fiducial marker was confirmed via CT. After the sheath was removed, patients were required to rest for 4 h to stop the bleeding. Irrespective of the placement method, patients without serious complications were discharged the next day and began proton therapy ~10 days later.

### Proton therapy system

Proton therapies were delivered by PROBEAT III (Hitachi, Ltd, Tokyo, Japan) and planned with VQA (Hitachi, Ltd). All treatments for pancreatic cancer employed a passive scattering technique with mainly 120–200 MeV proton beams. For treatment planning, patients were immobilized in the supine position, and 2-mm-thick CT slices were taken during the expiration phase. All patients underwent four-dimensional CT (4D-CT) to account for tumor motion with deformation. Patient respiratory waveforms were monitored throughout the procedures and recorded with an AZ-733 V respiratory gating system (Anzai Medical, Tokyo, Japan). Daily patient alignments were achieved using fluoroscopy. After bone matching, fiducial marker matching was performed to correct the position. All image-guidance procedures were performed with 2D/2D matching methods using the PIAS system (Hitachi, Ltd, Tokyo, Japan) [14, 15].

### Assessment

The following patient characteristics were compared between percutaneous and transarterial fiducial marker placement: sex, age, size and location (head/body/tail) of pancreatic cancer. Technical success was defined as the reliable use of the placed fiducial marker throughout the entire proton therapy course. Before proton therapy, 4D-CT was used to confirm that the tumor and fiducial marker were synchronized during breathing. After that, at each treatment, an X-ray was taken at maximum expiration to check whether the marker position had changed significantly from the previous time. The following were considered technical failures: the marker had moved or there was poor respiratory synchronization at the time of 4D-CT, or the marker position changed significantly during treatment, and subsequent CT scans determined that there was poor synchronization with the tumor, leading to a change to another matching method. Since we do not monitor the markers in real time during irradiation, we cannot verify mis-synchrony in real time with our equipment. If we observe any changes in the marker position in the interfractional region during the treatment course, we change from marker matching to bone matching and respond by frequently checking with CT scans. Major complications were defined as grade 3 or higher based on ‘Common Terminology Criteria for Adverse Events, version 5’. Minor complications (those not requiring invasive treatment) were defined as grade 2 or lower on ‘Common Terminology Criteria for Adverse Events, version 5’. The procedure time for percutaneous placement was measured as the interval from needle insertion to marker placement, and that for transarterial placement was measured as the interval from sheath insertion into the right femoral artery after local anesthesia to marker placement. The distance between the tumor and the placed fiducial marker was measured by creating multi-planar reconstruction from planning CT with contrast media taken at least 1 day after placement. Measurements were performed by a diagnostic radiologist with 9 years of relevant work experience using diagnostic software (EV Report ver. 4.0.0.11367, PSP Corporation, Tokyo, Japan). If the marker was located within the tumor or on the surface, the distance between the tumor and the marker was set to 0 mm. If there were contraindications to the use of contrast media, non-contrast CT was used to detect tumors. For percutaneous placement, the following categories were classified according to the puncture route (ventral or dorsal), organs in the puncture path, situ of fiducial marker placed. For transarterial placement, categories were classified according to the vessel in which the marker was placed. Each outcome was compared between percutaneous and transarterial placement using Fisher’s exact test or the Mann–Whitney U test. Spearman’s rank correlation coefficient was evaluated between tumor-marker distance and the incidence of synchronization failure.

All *P*-values were two-sided, and *P* ≤ 0.05 was considered statistically significant. All statistical analyses were performed with EZR (Saitama Medical Center, Jichi Medical University, Saitama, Japan), a graphical user interface for R (The R Foundation for Statistical Computing, Vienna, Austria, version 4.3.1). More precisely, it is a modified version of R commander (version 2.9-1) designed to add statistical functions frequently used in biostatistics [16].

## RESULTS

**Fig. 1:**
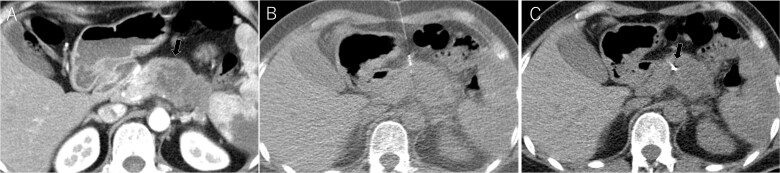
A 53-year-old male with pancreatic cancer. (**A**) Contrast-enhanced CT shows pancreatic cancer at the pancreatic body and tail (arrow). (**B**) Percutaneous fiducial marker placement was performed. The needle was inserted through the ventral side of the pancreatic cancer. (**C**) Postprocedural CT shows an implanted fiducial marker beside the pancreatic cancer without migration (arrow).

**Fig. 2:**
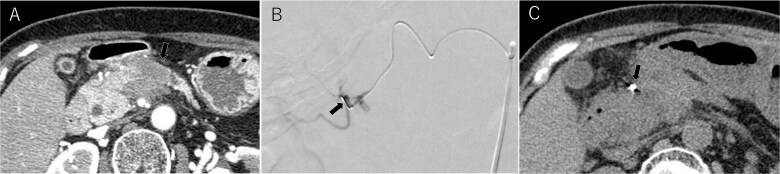
A 76-year-old female with pancreatic cancer. (**A**) Contrast-enhanced CT shows pancreatic cancer at the pancreatic body (arrow). (**B**) A branch of the anterior superior pancreaticoduodenal artery near the tumor was selected using a microcatheter, and a straight coil was placed as a fiducial marker (arrow). (**C**) Postprocedural CT shows an implanted fiducial marker beside the pancreatic cancer without migration (arrow).

There were 159 patients planned for proton therapy for pancreatic cancer during the applicable period. Of these, 110 were eligible. In one patient, duodenal invasion was observed during the examination after marker placement, and proton therapy was discontinued. Of the remaining 109 patients, 49 underwent percutaneous fiducial marker placement and 60 underwent transarterial fiducial marker placement (Figs 1 and 2).

There were no statistically significant differences in the sex ratio, age and the sizes and locations of pancreatic cancer (head/body/tail) between the two groups (Table 1). In percutaneous placement, the needle was inserted through the ventral side in 37 out of 49 cases, via the liver in 14 out of these, and the marker was placed within the tumor in 24 cases. In transarterial placement, the most common location was in the anterior superior pancreaticoduodenal artery, occurring in 28 out of 60 cases (Table 2). The markers were folded in all cases for percutaneous placement, and straight in all cases for transarterial placement.

**Table 1:** Percutaneous versus transarterial placement

	Percutaneous	Transarterial	
No. of patients (male/female)	49 (22/27)	60 (27/33)	*P* > 0.99^*^
Age^a^	73 [50–88]	71 [47–90]	*P* = 0.08^**^
Size of pancreatic cancer (mm)^a^	33 [16–86]	35 [19–69]	*P* = 0.90^**^
Location (head/body/tail)	16/16/17	24/16/20	*P* = 0.67^*^
Technical success	89.8% (44/49)	90.0% (54/60)	*P* > 0.99^*^
Major complication	0.0% (0/49)	0.0% (0/60)	*P* > 0.99^*^
Minor complication	2.0% (1/49)	5.0% (3/60)	*P* = 0.62^*^
Procedure time (min)^a^	3.3 [1.2–15.5]	40.0 [15.0–95.0]	*P* < 0.01^**^
Distance between tumor and marker (mm)^a^	0.0 [0.0–50.0]	10.0 [0.0–55.0]	*P* < 0.01^**^
No. of fiducial markers (1/2/3/4/0)	47/2/0/0/0	53/5/0/1/1	*P* = 0.68^*^

^a^Quantitative data are presented as the median. The square brackets indicate the range (from the minimum to the maximum value).

^*^Fisher’s exact test.

^**^The Mann–Whitney U test.

**Table 2:** Details of percutaneous and transarterial fiducial marker placement

Fiducial marker placement	
**Percutaneous (*n* = 49)**	
**Approach**	
Ventral/Dorsal	37/12
**Via organs**	
Non/Liver	35/14
**Location of the fiducial marker**	
In tumor/pancreatic parenchyma/ventral to the pancreas/dorsal to the pancreas	24/9/7/9
**Transarterial (*n* = 60)**	
**Artery with a fiducial marker placed**	
ASPD/PSPD/TP/DP/GP/Others	28/11/6/5/5/5

ASPD = anterior superior pancreaticoduodenal artery, PSPD = posterior superior pancreaticoduodenal artery, TP = transverse pancreatic artery, DP = dorsal pancreatic artery, GP = great pancreatic artery.

The technical success rate was 89.8% (44/49) for percutaneous fiducial marker placement and 90.0% (54/60) for transarterial placement with no significant difference between the two rates (*P* > 0.99). There were five cases of technical failure in percutaneous placement, and in three of these cases, respiratory synchronization between the tumor and marker failed on 4D-CT before proton therapy (Table 3). In these three cases, the fiducial marker was suspected to be beneath the anterior renal fascia, two of which were punctured dorsally and one ventrally. In the other two cases in which fiducial markers were placed from the ventral side, the coil migration to the abdominal cavity was observed. Therefore, the migration rate for percutaneous placement was 4.1%. The marker was stable just after placement; however, CT at a later date revealed migration. Additional markers were not placed to prevent proton therapy delay. These cases were converted to bone matching. In two cases of percutaneous placement, there was erroneous puncture of an artery and the marker migrated to the peripheral artery; in one case, this was the hepatic artery running through the tumor, and in the other, it was the splenic artery. There was no bleeding in either case. In the former case, the migrated marker was used as a fiducial marker, and in the latter case, another marker was placed. Technical success included these two cases because the markers were able to be used until the end of proton therapy. There were six cases of technical failure with transarterial placement; three of them were cases of failure of synchronization between tumor and marker during breathing, two of them were cases of marker migration to the distal vessel, and one was a case in which the marker could not be placed due to the complexity of the artery after surgery. Two cases of tumor-marker synchronization failure were diagnosed during proton therapy and were converted to bone matching. The migration rate for transarterial placement was 3.3%, which was not significantly different from the 4.1% for percutaneous placement (*P* = 0.81, Fisher’s exact test). Any fiducial markers did not migrate during proton therapy in both groups.

**Table 3:** Technical failures and minor complications

	Percutaneous		Transarterial
	Ventral 37	Dorsal 12	60
**Technical failures**			
Synchronization failure of the marker and tumor during proton therapy	1	2	3
Migration to the abdominal cavity	2		
Migration to a distal artery			2
Failure to place the marker			1
**Minor complications**			
Mesenteric microbleeds	1		
Dissection of a peripheral artery			1
Nausea			1
Subcutaneous hematoma in the right femoral region			1

There was no major complication with either technique, and pancreatitis did not occur. As a minor complication, minor mesenteric bleeding was observed in one case of percutaneous placement; however, the bleeding stopped spontaneously soon after. In one case of transarterial placement, the dissection of the posterior superior pancreaticoduodenal artery due to catheter manipulation was observed; however, the patient was treated conservatively.

The median procedure time was 3.3 min (range; 1.2–15.5) for percutaneous placement and 40.0 min (range; 15.0–95.0) for transarterial placement. The percutaneous placement was significantly shorter (*P* < 0.001). The median distance between the pancreatic tumor and the placed fiducial marker was 0.0 mm (range; 0.0–50.0) for percutaneous placement and 10.0 mm (range; 0.0–55.0) for transarterial placement. The percutaneous placement was significantly shorter (*P* = 0.005). Spearman’s rank correlation coefficient for the tumor-marker distance and the incidence of synchronization failure was *ρ* = −0.286 for percutaneous placement and *ρ* = −0.265 for transarterial placement, both of which showed a weak correlation.

## DISCUSSION

We found a high success rate for percutaneous fiducial marker placement for pancreatic cancer, equivalent to that for transarterial placement. This success rate is comparable to that of the transarterial placement reported by Imaizumi *et al*. [8]. There were no major complications with both methods. For percutaneous fiducial marker placement, puncture was performed using a thin-bore needle (25G). Complications are unlikely to occur because the needle is thinner than the 20G or 22G needles commonly used in endoscopic ultrasound-guided fine needle aspiration [17]. In fact, percutaneous transgastric pancreatic biopsy using needles larger than 18–22G has been reported, and most studies have not reported any serious complications [18–20]. Therefore, even in cases where percutaneous fiducial marker placement from the dorsal side is difficult, placement from the ventral side has been made possible. In this study, ventral-side puncture was performed in 37 out of 49 cases. This demonstrates sufficient feasibility. However, given the sample size, this finding may reflect limited statistical power rather than true equivalence in safety. Rare but clinically important complications may not be adequately captured in this study.

The procedure time was much shorter with percutaneous placement than with transarterial placement. This method is suitable for placing fiducial markers in patients with pancreatic cancer, who are becoming more numerous, and it allows more patients to be treated in a short period. Compared to transarterial placement, percutaneous placement is expected to impose less physical burden on patients. But, in this study, direct evaluation of invasiveness was not performed because pain scales and other data were not collected prospectively. Further study should be necessary.

The distance between the pancreatic tumor and the placed fiducial marker was significantly shorter with percutaneous placement than with transarterial placement. For the latter, it was a little difficult to select a peripheral artery near the tumor because of the pancreatic artery’s small caliber. Although the placement of a metal marker in the tumor has some effect on the radiation dose calculation, we placed some importance on ensuring a more accurate irradiation range. In percutaneous placement, markers were placed in a folded form in all cases, but Uludag reported that folding markers have little effect on proton beam dose reduction [21]. At our own institution, we have confirmed through Monte Carlo simulation that the foldable markers used in proton therapy for prostate cancer have little effect on uncertainty in the proton beam range and reduced irradiation accuracy. It has also been reported that multiple-field irradiation reduces the attenuation of the proton beam due to shadowing by the marker [22], and this method was also used in the pancreatic cancer cases in this study.

Seeding by tumor puncture was not confirmed in this study. On the other hand, migration occurs when a marker cannot be placed in the tumor by percutaneous puncture. When puncturing from the ventral side, it is important to avoid dropping the marker into the abdominal cavity. However, it was hard to distinguish the pararenal space from the abdominal cavity on CT. Therefore, two cases in which the marker fell off into the abdominal cavity were revealed by CT the day after percutaneous placement. To avoid migration to the abdominal cavity, the marker should be placed in the pancreatic tumor or the pancreatic parenchyma.

There was no migration with dorsal puncture; however, it is important to take care not to mis-synchronize the tumor and the fiducial marker during proton therapy. All two cases of technical failure of dorsal puncture were synchronization failure of the marker and tumor during proton therapy because the marker was placed beneath the anterior renal fascia. Although the pancreas is an organ whose location exhibits relatively large fluctuations during respiration [23], these markers did not move as much as pancreatic tumors and could not function as fiducial markers. This is because tissues dorsal to the anterior pararenal space are relatively fixed and exhibit little respiratory variability [24–26]. Therefore, when placing a fiducial marker outside the pancreas, dorsal puncture requires careful placement to prevent synchronization failure. One of the important conditions for a fiducial marker to be useful in radiotherapy is that it must be close to the tumor [27]. Another important condition is that the pancreatic tumor and the marker must move as synchronously as possible during breathing [28]. This study suggested a weak correlation: the further the marker was placed from the pancreatic tumor, the higher the incidence of synchronization failure. Therefore, the fiducial marker should be placed near the tumor. However, our proton beam system does not allow for real-time confirmation of the tumor and marker positions, which limits the assessment of these items. The clinical implications of this are that the set margins may not be able to accommodate unexpected target movement during irradiation, resulting in the target dose not being maintained. One of the preventive strategies against this is to use Real-time Gated Proton Therapy. Real-time monitoring during irradiation is a useful tool [29]. In addition, extrapancreatic placement is expected to be the main cause of mis-synchronize, and measures include placing the marker inside the pancreas or tumor as much as possible, and placing multiple markers if they appear to be placed outside the pancreas.

Percutaneous puncture of the pancreas is useful but often difficult. This is because the pancreas is surrounded by other organs and has many important blood vessels around it, making it difficult to avoid them. The second reason is that the pancreatic parenchyma is often atrophied in patients with pancreatic cancer and elderly people, shrinking the target. Another reason is that the pancreas moves a lot with breathing. As mentioned above, movement of the tumor and marker during respiration must also be considered. Puncture of the pancreas requires a certain level of skill, and it should be performed by an experienced interventional radiologist.

This puncture placement technique was performed for proton therapy; however, it is also thought to be useful for image-guided radiation therapy and stereotactic irradiation using photons which have been on the rise in recent years.

Nevertheless, this study has some limitations that are worth mentioning. The first is a statistical problem. This study was conducted as a single institution retrospective study. Although there were no obvious imbalances in baseline patient characteristics, it is difficult to fully exclude the influence of potential confounding factors not evaluated in this study. Furthermore, due to the decision-making process for the placement method, there may have been a relatively higher number of cases in which percutaneous placement was easier to perform. There is a possibility of patient selection bias. Second, all procedures were performed by experienced interventional radiologists at a single institution. The feasibility and safety reported in this study may therefore not be directly generalizable to centers with different levels of expertise or procedural volume. Another limitation was the problem of how to measure time. Although time was measured in terms of procedure time, it might have been more appropriate to measure the time spent in the procedure room; however, this parameter was not recorded.

In conclusion, percutaneous fiducial marker placement for pancreatic cancer proton therapy using a 25G needle could be performed faster than transarterial placement with a high success rate.

## PRESENTATION AT A CONFERENCE

CIRSE 2024.

## Clinical Trial Registration number

The institutional review board of Nagoya City University Hospital approved this study (No. 60-24-0041).
